# Mouse developmental defects, but not paraganglioma tumorigenesis, upon conditional Complex II loss in early Sox10^+^ cells

**DOI:** 10.1096/fba.2024-00056

**Published:** 2024-07-24

**Authors:** Elizabeth P. Lewis, Fatimah Al Khazal, Brandon Wilbanks, Naomi M. Gades, Patricia Ortega‐Sáenz, José López‐Barneo, Igor Adameyko, L. James Maher

**Affiliations:** ^1^ Department of Biochemistry and Molecular Biology Mayo Clinic College of Medicine and Science Rochester Minnesota USA; ^2^ Department of Comparative Medicine Mayo Clinic Scottsdale Arizona USA; ^3^ Instituto de Biomedicina de Sevilla Hospital Universitario Virgen del Rocio/CSIC/Universidad de Sevilla Sevilla Spain; ^4^ Department of Physiology and Pharmacology Karolinska Institutet Solna Sweden; ^5^ Department of Neuroimmunology, Center for Brain Research Medical University of Vienna Vienna Austria

**Keywords:** familial paraganglioma, gait defect, melanocyte, mouse, neural crest, pheochromocytoma, Sox10, succinate dehydrogenase

## Abstract

In humans, loss of heterozygosity for defective alleles of any of the four subunits of mitochondrial tricarboxylic acid cycle enzyme succinate dehydrogenase (SDH, also Complex II of the electron transport chain) can lead to paraganglioma tumors in neuroendocrine cells. With the goal of developing mouse models of this rare disorder, we have developed various SDH conditional loss strategies. Based on recent lineage tracing studies, we hypothesized that conditional SDHC loss in early embryogenesis during migration of primordial neural crest cells that form the susceptible chromaffin cells of the adrenal medulla might induce paraganglioma. We triggered low levels of detectable SDHC loss in Sox10^+^ cells at E11.5 of mouse development. We report that, rather than developing adrenal medulla paraganglioma (pheochromocytoma), offspring survived with evidence of neural crest cell dysfunction. Phenotypes included mild lower extremity gait anomalies suggestive of neural tube closure defects and patches of unpigmented fur consistent with neural crest‐derived melanocyte dysfunction. These defects were not observed in mice lacking *Sdhc* knockout. Our results add to existing data suggesting that, unlike humans, even early embryonic (Sox10‐driven) SDHx loss is inadequate to trigger paraganglioma in mice of the genetic backgrounds that have been investigated. Instead, low levels of tricarboxylic acid cycle‐deficient neural crest cells cause mild developmental defects in hind limb and melanocyte function. This new model may be of interest for studies of metabolism during early neural crest cell development.

## Graphical abstract

In humans, paragangliomas (PGLs) form in certain neuroendocrine cells that have lost succinate dehydrogenase, an enzyme of the tricarboxylic acid cycle. Conditional SDH deficiency has not yet been shown to drive PGL formation in mice, limiting available research tools. Here, prenatal SDH knockout in small numbers of SOX10+ precursors of adrenal medulla chromaffin cells was attempted. PGL was not observed, but other neural crest developmental defects were seen, including effects consistent with melanocyte dysfunction and neural tube closure defects.

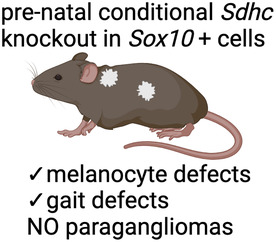

## 1 INTRODUCTION

Mutations affecting mitochondrial energy production pathways often present as inborn errors of metabolism leading to a variety of autosomal recessive disorders such as Leigh syndrome.^1^, ^2^ Paradoxically, in other contexts similar defective variants predispose with dominant genetics to hereditary tumors such as gastrointestinal stromal tumor (GIST) and pheochromocytoma and paraganglioma (PPGL).^3^, ^4^, ^5^ About 40% of PPGL tumors result from loss of heterozygosity (LOH) uncovering germline tumor suppressor variants promoting a pseudohypoxic phenotype. Such variants include nuclear DNA mutations that inactivate any of the four subunits (A–D) of succinate dehydrogenase (SDH) in the tricarboxylic acid (TCA) cycle.^6^ Among mechanisms proposed to link SDH loss to PPGL tumorigenesis, a compelling model is the accumulation of succinate as an oncometabolite.^7^ Succinate accumulation induces downstream pathologies including inhibition of 2‐ketoglutarate‐dependent dioxygenases involved in the normal degradation of hypoxia‐inducible factors and demethylation of histones, DNA, and RNA.^7^, ^8^, ^9^, ^10^ The resulting dysregulation of metabolism and epigenetics is apparently tumorigenic in certain tissues.^11^, ^12^, ^13^ PPGL tumors arise in neuroendocrine cells including the carotid bodies (CBs), which originate from neural crest progenitor cells.^14^ There is evidence that it is the glomus type I neuron‐like cells of the CB that are susceptible to PGL tumorigenesis.^15^ These cells express tyrosine hydroxylase (TH).^16^ Related cells susceptible to tumorigenesis after SDH loss are paraganglia^17^ and the interstitial cells of Cajal.^18^

Despite multiple attempts to develop mouse models of SDHx‐deficient hereditary PPGL, no successes have been reported.^9^, ^19^, ^20^, ^21^, ^22^, ^23^, ^24^, ^25^ As in humans, homozygous SDH loss in mice is an embryonic lethal condition. Unlike humans, heterozygosity for loss‐of‐function *Sdhx* variants does not predispose mice to PPGL. Conditional *Sdhx*‐knockout strategies have been attractive to target SDHx loss to certain vulnerable cell populations and developmental stages to test the hypothesis that PPGL tumor initiation can be achieved by acceleration of SDH loss. We previously triggered SDHC loss selectively in susceptible TH^+^ neuroendocrine cells including cells of the central nervous system, paraganglia, the adrenal medulla, and the glomus cells of the CB.^24^ That design employed a TH promoter‐driven Cre recombinase to induce deletion of SDHC in TH^+^ cells. Additional tumor promotion strategies (chronic 10% oxygen hypoxia and conditional expression of a dominant negative p53 tumor suppressor protein) were also tested for their impacts on PPGL tumorigenesis in this model. None of these conditions resulted in PPGL. Surprisingly, SDHC loss in TH^+^ cells caused an obesity phenotype starting at 20 weeks of age.^24^ We hypothesized that this phenotype results from the deletion or altered behavior of SDHC‐loss TH^+^ cells important for dopamine signaling in feeding behavior and obesity.^26^, ^27^

Here, we describe a refined approach to conditional SDH knockout. We targeted SDH loss to neural crest‐derived Sox10^+^ cells early in mouse embryonic development in an attempt to elicit PPGL tumorigenesis specifically in the adrenal medulla. This strategy is based on published lineage tracing^28^, ^29^ suggesting that during a developmental window of E9.5–E11.5, PPGL‐vulnerable neural crest‐derived adrenal medulla precursor cells are arriving at the developing adrenal gland of the mouse. We hypothesized that SDH loss at this time and in this compartment would lead to PPGL tumorigenesis through mechanisms under study.^28^, ^30^ We developed an approach to trigger prenatal *Sdhc* knockout in deliberately low numbers of migrating neuroendocrine cells between E9.5 and E11.5. This was accomplished by i.p. administration of moderate tamoxifen (TAM) doses to gravid female mice carrying *SdhC* ^*fl/fl*^ embryos, some of which also carried *Sox10‐iCreER* ^*T2*^ driver genes activated by TAM.

We show that timed moderate TAM dosing of gravid females drives the desired low, but detectable, iCRE‐dependent *Sdhc* loss in utero in Sox10^+^ tissues of developing embryos. The rationale for moderate TAM dosing was to trigger knockout rearrangement of floxed *Sdhc* genes in a low but detectable number of Sox10^+^ cells to test the hypothesis that each resulting SDH‐null adrenal medulla precursor cell might seed a PPGL tumor. We sought to avoid metabolically damaging entire embryonic tissues while preserving normal gestation and delivery by gravid females without the need for cesarean section and fostering.

We report that the intended spaciotemporal *Sdhc* knockout was achieved at E11.5 in the developing adrenal medulla but with no evidence for PPGL tumorigenesis. Interestingly, affected mice commonly displayed a moderate hind limb gait defect suggesting the possibility of minor neural tube closure defects. *Sdhc* conditional knockout also often displayed a distinctive pattern of white fur patches suggesting that ablating oxidative phosphorylation in a subset of Sox10^+^ cells of neural crest‐derived melanocytes prevented pigmentation.

## 2 MATERIALS AND METHODS

### 2.1 Ethical treatment of animals

All experimental procedures performed in this study were approved by the Mayo Clinic Institutional Animal Care and Use Committee (IACUC protocol numbers A00005367 and A00004588) in accordance with NIH guidelines. Animals were transferred to the experimental protocol at weaning and observed for a total of 18 months before sacrifice unless humane endpoint criteria were met at an earlier time.

### 2.2 Generation of SDHC conditional knockout mice

The *Sdhc* gene‐trap line C57BL/6N‐*Sdhc* ^*tm1a(EUCOMM)Wtsi*^ was obtained from the European Conditional Mouse Mutagenesis Program at the Sanger Center, UK. These animals carrying FLP‐FRT recombination signals targeting exon 4 of *Sdhc* were crossed with flippase recombinase‐expressing mice to produce an *Sdhc* floxed (*fl*) allele (Figure S1). These mice were then crossed with *Sox10::iCreER* ^*T2*^ mice^31^ (the generous gift of Vassilis Pachnis). *Sdhc* genotyping primers were LJM‐4429 (CT_2_AGA_2_CTGATC_4_TGC_3_), LJM‐4430 (CACTGC_3_G_3_CTCATAT_3_C), and LJM‐5125 (C_2_TG_2_A_2_CTAGA_2_T_2_AT_2_GATG_2_ATG). *iCreER* ^*T2*^ genotyping primers were LJM‐6242 (T_2_GCGATG_3_AGAGTCTGAC) and LJM‐6243 (AG_2_TACAG_2_AG_2_TAGTC_3_TC). *Sdhc* genotyping was with primers 4429 + 4430 (yielding a diagnostic 411‐bp WT product or a 595‐bp *fl* PCR product, or with primers 4429 + 5125 yielding a 560‐bp knockout PCR product; Figure S1). The desired experimental mice were *Sdhc* ^*fl/fl*^; *Sox10::iCreER* ^*T2*^. Controls were *Sdhc* ^*fl/fl*^.

### 2.3 Mouse husbandry

Five or fewer mice were housed with same‐sex littermates in filter‐capped polycarbonate cages supplied with PicoLab Rodent Diet 20 chow and filter‐purified tap water ad libitum in a room with constant temperature (21 ± 2°C) and humidity (45 ± 10%). Animals were exposed to a day–night cycle of 12 h.

### 2.4 Triggering low levels of *Sdhc* knockout in Sox10^+^ cells by TAM dosing of gravid females

Mice were caged on cubical racks in an isolated room to reduce foot traffic and stress. The Whitten effect was applied to synchronize female estrus cycles. Isopads (Braintrain Scientific, Inc.; ISO‐6105) were infused with male pheromones by bedding for at least 24 h. Groups of 1–4 females were then introduced to pheromones from a single male by exposure to a soiled isopad 3 days prior to mating. Females were then introduced to males for 24 h. After this mating interval, females were examined for the presence of a vaginal plug and separated from the male. Females presenting a vaginal plug were weighed and placed into single housing. These females were scheduled for TAM dosing, and the date was noted as E0.5. Timed TAM dosing was performed at E9.5, E10.5, or E11.5. Females received intraperitoneal injections of sterile filtered TAM (dissolved in corn oil) at a dose of 17 μg/g body weight. This moderate dose of TAM was employed with the goal of *Sdhc* knockout in a fraction of adrenal cells while allowing for gestation and parturition. Delivered mice were weaned and transferred to IACUC protocol A00005367 for monitoring and aging.

### 2.5 Animal aging

Mice were observed three times weekly and euthanized promptly if IACUC humane criteria were met, such as when extreme weight loss, tumor growth, or a hunched posture were observed. Healthy mice were aged to 18 months before sacrifice and tissue fixation by perfusion. Animals were examined weekly for evidence of tumors.

### 2.6 Pathology

All carcasses were examined for gross evidence of tumors at sacrifice. Carotid body tissues from a subset of animals were fixed, sectioned, and stained with hematoxylin and eosin, and subjected to immunohistochemistry for tyrosine hydroxylase to monitor chromaffin cells. Adrenal glands, skin, and spinal cords from an additional subset of animals were sectioned and reviewed after hematoxylin and eosin staining. The following procedures were used for immunofluorescence staining by antityrosine hydroxylase and anticytochrome C or antityrosine hydroxylase and anti‐SDHC at the Pathology Research Core (Mayo Clinic, Rochester, MN, USA) using a Leica Bond RX stainer (Leica). Tissue sections were cut at 5 μm, mounted on charged slides, and dried overnight. Slides were baked for 30 min in a 60°C oven prior to loading onto a stainer. All slides were dewaxed, retrieved for 20 min using Epitope Retrieval 2 (EDTA based; Leica), and incubated in 10% Goat Serum Block (Thermo) for 30 min. Slides were incubated for 60 min in antityrosine hydroxylase (clone: EP1532Y; Abcam) antibody diluted at 1:250, followed by incubation for 60 min in goat‐antirabbit Alexa‐488 (Invitrogen) diluted at 1:400. For the following steps, slides were incubated for 60 min with either anticytochrome C conjugated to Alexa‐594 (clone: 7H8.2C12; Novus) or anti‐SDHC conjugated to Alexa‐594 (clone: C‐2; Santa Cruz) both diluted at 1:50. All antibodies were diluted using Background Reducing Diluent (Dako). Slides were incubated for 10 min in Hoechst 33342 solution (Invitrogen) at 1 μL/mL in distilled water. Slides were removed from the stainer and permanently mounted under cover slips using Antifade Prolong Gold (Invitrogen). Fluorescent images were obtained by tile scan on a Zeiss LSM780 AxioObserver microscope using a 10×/0.3 objective. Tiled images were collected in a 3 × 3 grid for a total area of 2.38 × 2.38 mm per combined set of tiles. CellProfiler was used to define adrenal medullae by tyrosine hydroxylase signal. Cytochrome C signal from these defined medullae regions was then measured. Sections stained with hematoxylin and eosin were imaged on an AxioScan Z1 slide scanner at 10× magnification.

### 2.7 Gait analysis

Gait abnormalities were monitored in a subset of E11.5 *Sdhc* knockout mice and controls using a Noldus CatWalk apparatus. Twelve mice (eight *Sdhc* knockout animals and four controls) were led down an enclosed walkway on a glass plate that recorded the movement of each mouse. Gait abnormalities differed between affected mice. Example pawprint data from individual mice were recorded.

## 3 RESULTS AND DISCUSSION

### 3.1 Generation of conditional SDHC‐loss mice

Inspired by the goal of creating a mouse model of the rare familial neuroendocrine tumor PPGL,^20^ we utilized a gene trap approach to create a null *Sdhc* allele that, when rearranged by Flp recombination, created a conditional floxed *Sdhc* allele where two *LoxP* cites flank exon 4 as described (Figure S1).^23^ When exposed to Cre recombinase, exon 4 is deleted, yielding a mRNA encoding a nonfunctional, truncated SDHC protein (Figure [Link], [Link], [Link]). To avoid the embryonic lethality associated with systemic deletion of *Sdhc* in homozygous floxed animals,^20^ we knocked out *Sdhc* expression at deliberately low levels in an attempt to initiate clones of transformed PPGL cells. Prior approaches have been unsuccessful in mice.^20^, ^23^, ^24^, ^25^, ^32^ Based on new lineage tracing data showing that PPGL‐susceptible chromaffin cells of the adrenal medulla migrate from the neural crest along neurons to their final anatomical location in the adrenal medulla,^28^, ^29^ we hypothesized that SDHx loss must occur earlier in mouse development to generate PPGL.

We chose to use moderate levels (17 μg/g body weight) of TAM to trigger SDHC loss between embryonic E9.5–E11.5, seeking to avoid TAM‐induced developmental defects and delivery complications. We note that this TAM dose was tolerated by the current strain for successful delivery of conditional SDHC‐loss animals. We have recent evidence that other strain backgrounds may be more sensitive to this TAM dose.

**FIGURE 1:**
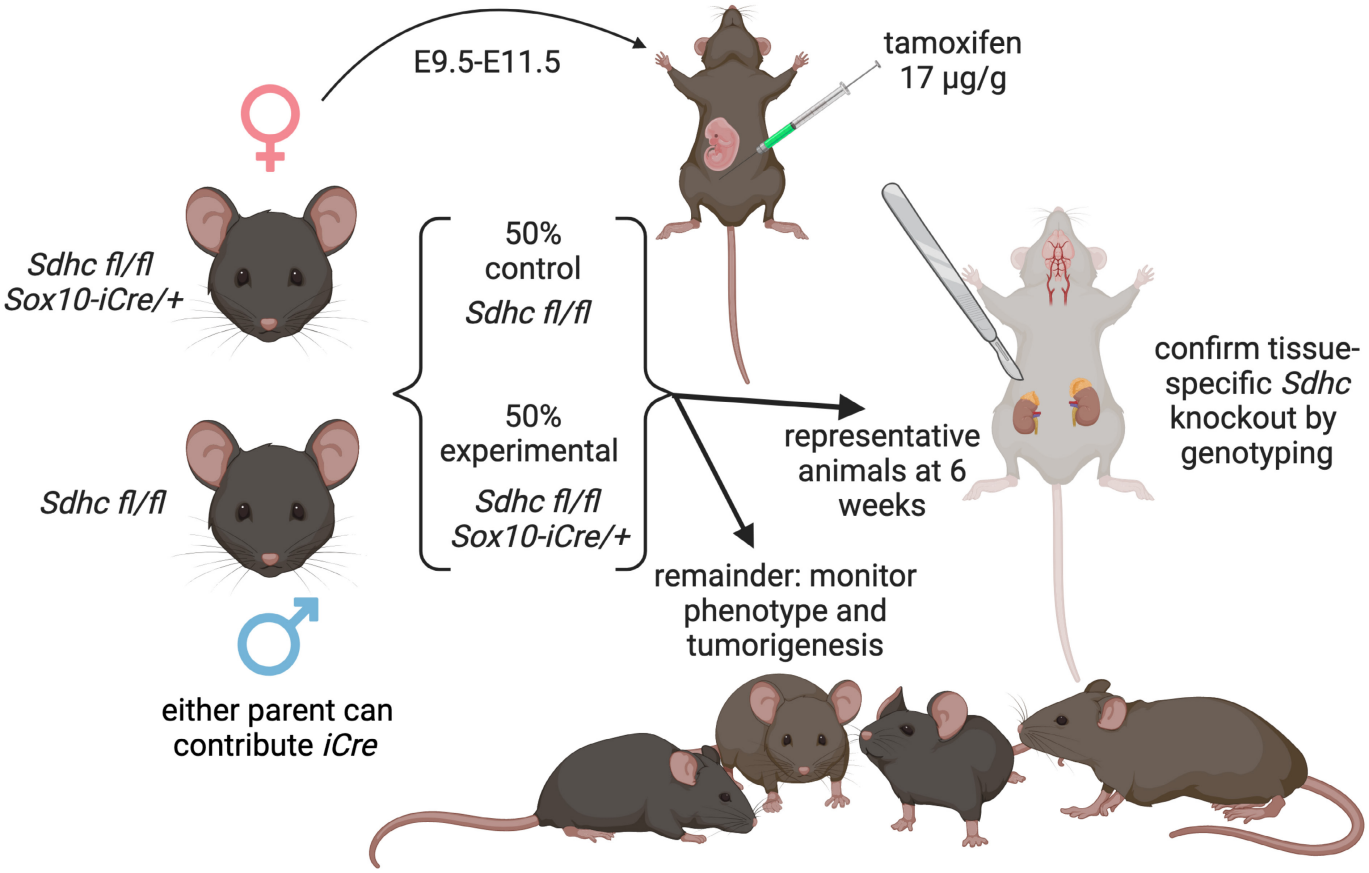
Experimental design. Conditional SDHC loss was triggered in utero by moderate doses of TAM injected i.p. into gravid females between E.9.5 and E11.5. This procedure targeted *Sox10* ^+^ cells of early *Sdhc* ^*fl/fl*^ embryos carrying *Sox10‐ER* ^*T2*^ *‐iCre* driver genes but not their *iCre* ^−^ littermates. At 6 weeks, representative animals were sacrificed and tissues genotyped for evidence of detectable *Sdhc* knockout. Remaining animals were aged and monitored for overt phenotypes and PPGL tumorigenesis.

**FIGURE 2:**
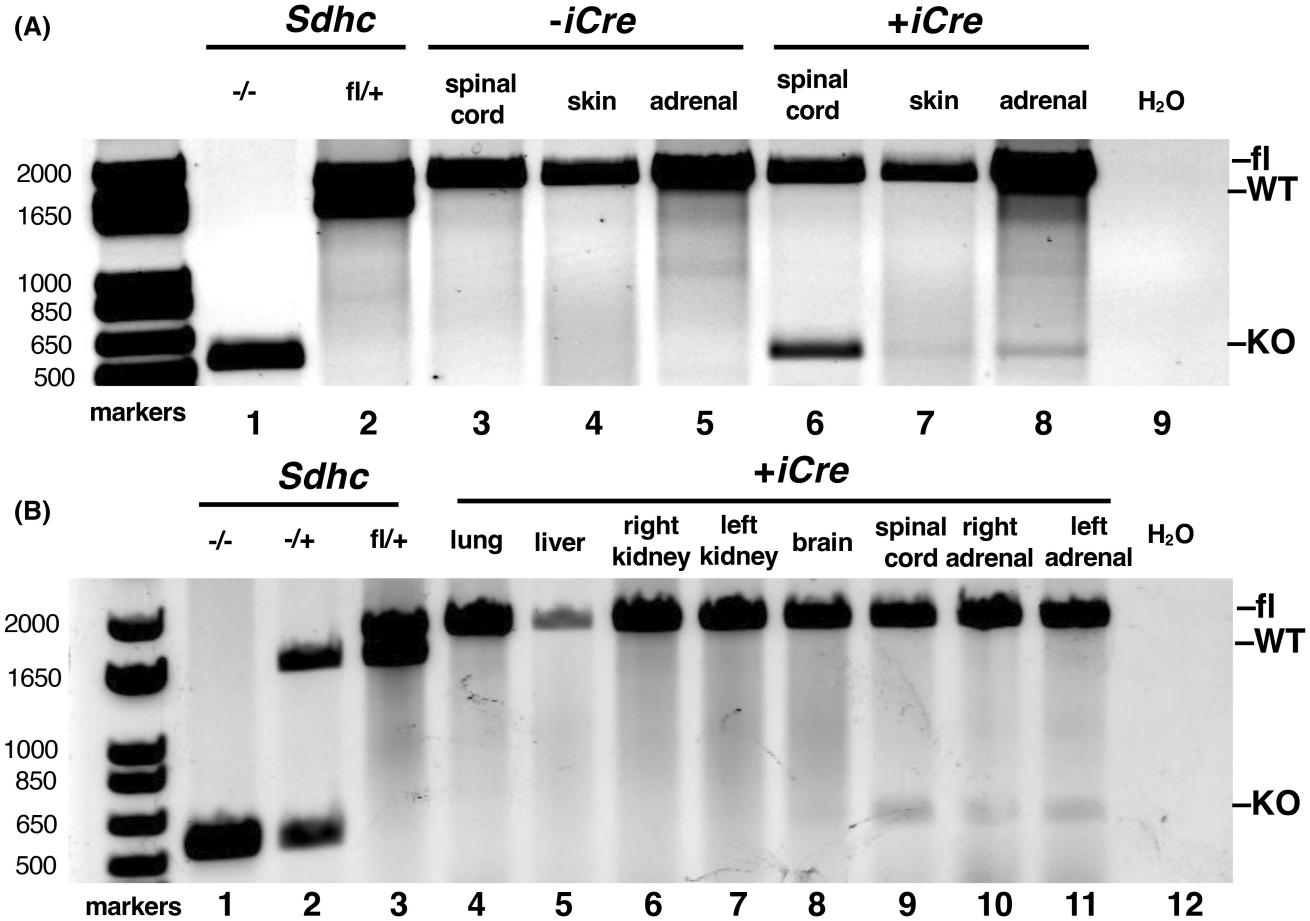
Confirmation of detectable tissue‐specific *Sdhc* knockout for littermates whose mother was injected with TAM at E11.5. (A) iCre specificity. Lanes 1–2, 9: *Sdhc* genotyping controls. Lanes 3–5: Absence of *Sdhc* knockout in the indicated tissues of mice lacking *iCre*. Lanes 6–8: Detectable tissue‐specific *Sdhc* knockout rearrangement in spinal cord and adrenal gland DNA, with traces of *Sdhc* rearrangement in skin. (B) Tissue specificity of *Sdhc* knockout for a *Sdhc* ^*fl/fl*^ *Sox10‐ER* ^*T2*^ *‐iCre* mouse whose mother was injected with TAM at E11.5. Lanes 1–3, 12: *Sdhc* genotyping controls. Lanes 4–11: Tissue‐specific *Sdhc* genotyping of the indicated tissues, with *Sdhc* knockout detectable in spinal cord and adrenal glands.

Our experimental approach is summarized in Figure 1. Mice carried two copies of the floxed *Sdhc* allele with or without the TAM‐inducible iCre‐ER^T2^ recombinase under the control of the neural crest‐specific *Sox10* promoter. We aimed to test the hypothesis that *Sdhc* ^*fl/fl*^ animals with sparse early embryonic knockout *Sdhc* would seed adrenal medulla PGL tumors (pheochromocytomas), while littermates lacking iCRE‐ER^T2^ would not. We generated mice with low levels of conditional SDHC loss as indicated by the tissue genotyping evidence shown in Figure 2.

### 3.2 No PPGL tumorigenesis in conditional SDHC‐loss mice

We used moderate TAM doses to conditionally knock out the SDHC subunit in Sox10^+^ cells at E9.5, E10.5, and E11.5 and surveyed the resulting experimental animals and their iCre^−^ littermate controls for anomalies and carotid body or adrenal medulla paraganglioma (pheochromocytoma).

Interestingly, TAM dosing at E9.5, E10.5, and E11.5 resulted in live‐born animals with PCR‐detectable SDHC knockout only at E11.5 (Figure 2). SDHC knockout was clearly detectable in spinal cord and adrenal gland tissue (Figure 2A, lanes 6 and 8) with suggestive evidence also in skin (Figure 2A, lane 7). More detailed tissue specificity of SDHC loss is shown in additional genotyping results (Figure 2B). Detectable *Sdhc* rearrangement is noted in spinal cord and adrenal gland (Figure 2B, lanes 9–11) but not in lung, liver, kidney, or brain (Figure 2B, lanes 4–8). No *Sdhc* knockout animals were recovered from E9.5 litters, and no gene rearrangement was detected among live births of *Sdhc* ^*fl/fl*^; *Sox10::iCreER* ^*T2*^ animals from gravid females injected at E10.5. We interpret these results to indicate that even partial SDHC loss at E9.5 was incompatible with embryogenesis, and only variably low TAM doses at E10.5 were tolerated for lives births, yielding undetectable levels of SDHC knockout in such offspring. Evidently, some of the E11.5 TAM doses were tolerated for embryogenesis, yielding detectable PCR rearrangement in tissues and full‐term delivery of *Sdhc* knockout pups.

No PGL tumorigenesis was observed in any of the 29 animals aged to 18 months. Microscopic pathology analysis of carotid bodies from 15 (mixed male and female) SDHC knockout animals and 2 controls showed no abnormalities. Likewise, microscopic adrenal gland pathology analysis of 12 mice (half male, half female, half iCre^+^, half iCre^−^) showed no PPGL tumorigenesis. Some medullae in both knockout and normal mice showed swollen coalescing cells with amorphous brownish material, presumed simply to be age‐related. A few knockout animals showed adrenal medullae with increased vascular spaces. We conclude that SDHC loss targeted to Sox10^+^ cells at E11.5 is not tumorigenic. Example of unaffected adrenal medullae from control and knockout animals with pigmentation and gait defects are shown in Figure S2.

**FIGURE 3:**
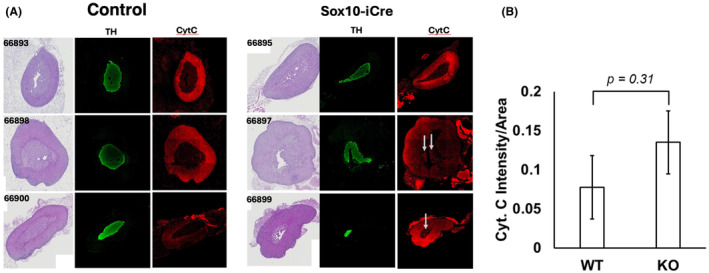
Immunofluorescent analysis of representative conditional knockout and control adrenal medullae. (A) Staining with hematoxylin and eosin (left column in each panel) or with antibodies against tyrosine hydroxylase (TH) or cytochrome C (CytC). Indices indicate specimen serial numbers. White arrows indicate evidence of enhanced cytochrome C expression within medullae, consistent with SDHC loss cells. (B) Quantitation of cytochrome C expression within tyrosine hydroxylase‐positive cells, showing a trend toward greater cytochrome C expression in *Sdhc* conditional knockout medullae, though this trend does not reach statistical significance (*t*‐test).

Although detectable tissue‐specific PCR detection of *Sdhc* rearrangement to generate knockout alleles and developmental phenotypes (see below) was evident, we also took steps to investigate whether the deliberately low levels of *Sdhc* knockout could be detected by histology in adrenal glands of conditional knockout animals and controls. We identified adrenal glands from three representative SOX10‐iCre cases that showed developmental phenotypes (see below) and three representative SOX‐10‐iCre‐negative cases without phenotypes. These specimens were sectioned and stained with hematoxylin and eosin, and the serial sections stained with antityrosine hydroxylase and anticytochrome C or antityrosine hydroxylase and anti‐SDHC (Figure S3). Definition of chromaffin cells by anti‐TH staining was effective. As anticipated, it was difficult to detect individual SDHC‐loss cells in knockout animals by this methodology. We therefore considered enhanced anticytochrome C staining as a surrogate for SDHC loss.^33^ The results are shown in Figure 3A, revealing a trend toward increased cytochrome C expression per unit area in adrenal medullae of SOX10‐iCre cases that had been treated with tamoxifen. The trend, though obvious and consistent with the appearance of individual cytochrome C positive cell clusters (arrows in Figure 3A), did not reach statistical significance (Figure 3B). This is not surprising to us in that the KO‐inducing tamoxifen dose was deliberately low, consistent with our goal of seeding PPGL tumors without tissue‐wide metabolic dysfunction. We conclude that *Sdhc* knockout in experimental adrenal medullae affected a small subset of cells as best detected by PCR.

### 3.3 Developmental defects in conditional SDHC‐loss mice

**FIGURE 4:**
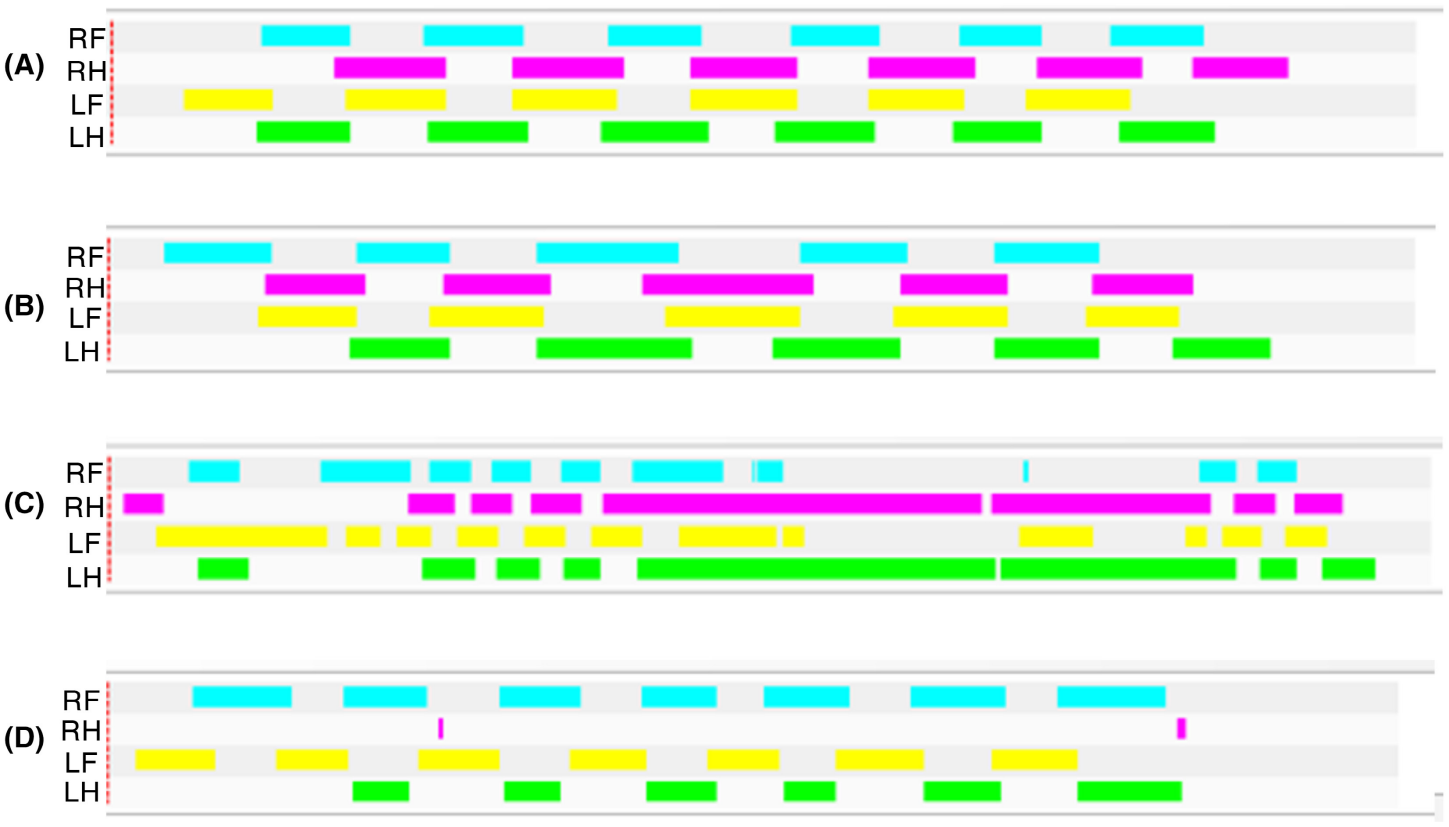
Abnormal gait of SDHC knockout mice analyzed by Noldus CatWalk instrumentation. Run images in timing mode are shown for two normal male control mice (traces A and B) and two male *Sdhc* knockout mice (traces C and D). In each trace, run timing is shown for the indicated paws: right front (RF), right hind (RH), left front (LF), and left hind (LH). Surface contact time for the indicated paw is recorded on the *x*‐axis (arbitrary units) allowing assessment of gait regularity. Missing signal suggests that the paw was not used for locomotion during this period. Sustained signal suggests that the paw was dragged along the surface.

Interestingly, while PPGL tumorigenesis was not observed, it was common that E11.5 TAM‐treated conditional *Sdhc* knockout experimental mice (but not controls) displayed one or both of two obvious phenotypes. One phenotype is a lower extremity gait defect (Videos [Link], [Link], [Link]). Animals demonstrated modest unilateral or bilateral hind limb paralysis. We used a Noldus CatWalk system to characterize this abnormality. Example run image data with timing is shown in Figure 4 for two control animals (traces A and B, and two SDHC knockout animals [traces C and D]). The gait defect in Figure 4 trace C shows evidence of paralysis and dragging of both hind legs, while the right hind leg does not create pawprints in Figure 4 Trace D. Gait defects were limited to the hind legs and were observed only in animals exposed to TAM at E11.5. Affected mice tended to hyperextend one or both hind legs, or locomote using the dorsal aspect of the ankle, foot, or hock rather than of the ventral aspect of the paw. We hypothesize that a neural tube closure defect might be involved in this gait abnormality.

**FIGURE 5:**
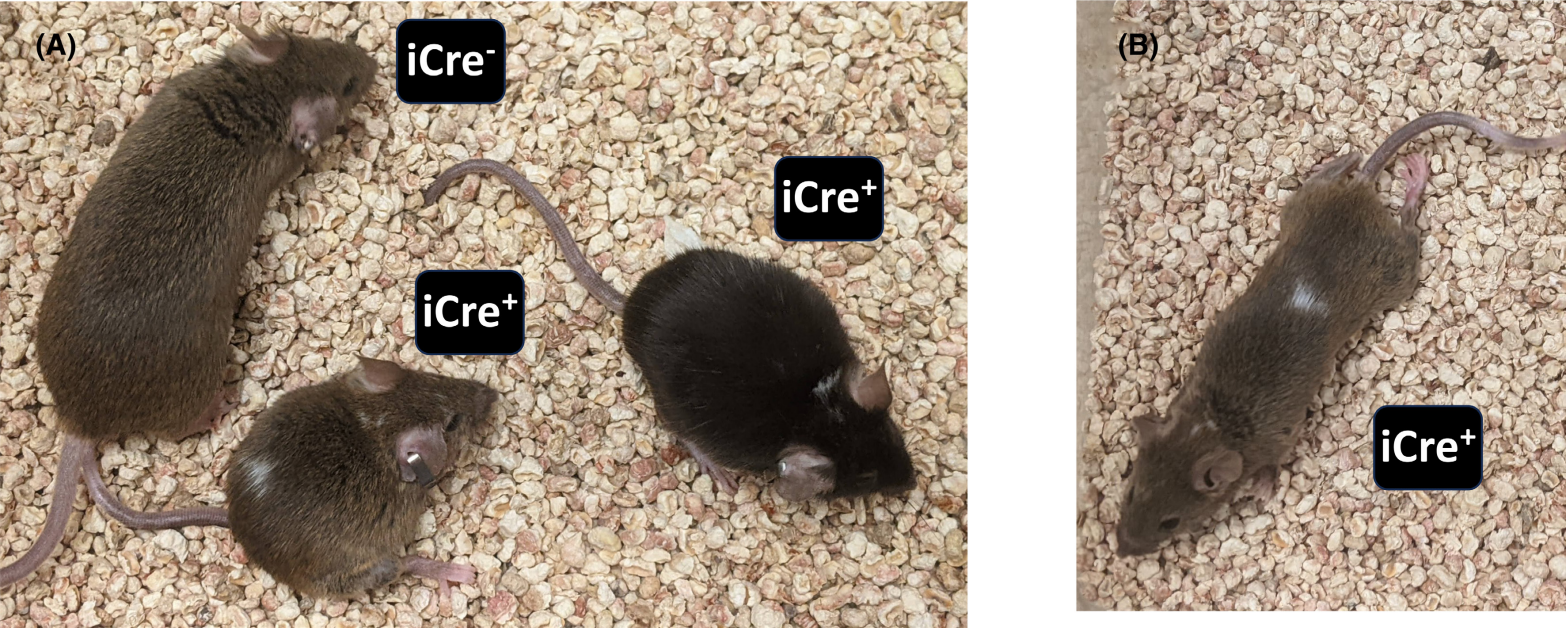
Morphological features of mice that sustained *Sdhc* knockout in Sox10^+^ cells triggered by moderate doses of TAM at E11.5. (A) Littermates of the indicated *iCre* status showing smaller size and characteristic patches of coat pigmentation defect. (B) An example of conditional *Sdhc* knockout animal triggered at E11.5 showing both a patch of unpigmented fur and hind limb gait abnormality.

The second phenotype (observed alone or in combination with the gait defect) was a pattern of patches of unpigmented fur (Figure 5). We hypothesize that clones of melanocytes (also a cell type derived from Sox10^+^ neural crest cells targeted for SDH loss) fail to develop proper pigmentation upon SDH loss during development.

## 4 CONCLUSIONS

We and others have been seeking to develop a mouse model of SDHx‐deficient familial PPGL. Such a model could have great value in studies of PPGL etiology and therapy. In human familial PPGL, heterozygosity for a loss‐of‐function *Sdhx* (particularly *Sdhb*) variant predisposes to PPGL with ~80% penetrance by age 70.^34^ The field lacks such mouse models of tumorigenesis and has only limited cell line models^8^, ^19^, ^20^, ^22^, ^35^, ^36^, ^37^ though recent progress has been reported in rat.^25^, ^38^ One hypothesis for the lack of SDH‐deficient PGL in mice is that the time required for spontaneous LOH in heterozygous SDH‐loss mice is too long for development of slow‐growing PGL tumors within the short lifespan of the animal. We and others therefore drive conditional SDH loss in tissues of interest. Whole‐body SDH loss is an embryonic lethal condition, though, remarkably, we have shown that conditional whole‐body SDH‐loss mice can survive in hypoxia.^23^ Others^33^ have successfully generated mice in which NF1 and SDHB were codeleted. Isolated deletion of SDHB caused an obesity phenotype that was similar to SDHC deletion, and codeletion of SDHB and NF1 resulted in development of SDHB‐deficient pheochromocytomas. H19 knockout was previously tested in combination with SDHD and was not confirmed to have a role in PPGL formation.

We hypothesized that tumorigenic SDHx loss might be required to occur at an earlier embryonic stage than heretofore modeled in mice. Tyrosine hydroxylase‐driven rearrangement occurs at and after E11 in development, and other artificial *Sdhx* conditional deletions have been triggered later after birth. Based on detailed lineage tracing, we set out to trigger low levels of SDHC loss in primordial Sox10^+^ neural crest cells shown to be precursors of the chromaffin cells of the adrenal medulla that are susceptible to PPGL tumorigenesis. Our results suggest that even low levels of SDHC loss in Sox10^+^ cells are not tolerated at E9.5 (no *iCRE‐ER* ^*T2*^ mice were born after TAM injection). At E10.5, *Sdhc* ^*fl/fl*^; *Sox10::iCreER* ^*T2*^mice were born, but none had detectable *Sdhc* rearrangement in the tissues of interest. Only in *Sdhc* ^*fl/fl*^; *Sox10::iCreER* ^*T2*^ mice from mothers injected with TAM at E11.5 were SDHC knockout cells detected in neural crest‐derived tissues. No PPGL tumorigenesis or tissue anomalies were observed among these animals.

It remains possible that an early developmental window for SDHx‐loss tumorigenesis was not adequately sampled in this study. Our TAM‐triggered SDHC loss around E11.5 in Sox10^+^ cells may overlap with previous unsuccessful attempts at PPGL tumorigenesis driven by TH‐iCre, likely inducing *Sdhc* knockout at similar time.^24^ It is also possible that the reportedly more penetrant SDHB‐loss condition could be required for PPGL tumorigenesis in mice. These studies are ongoing, though complicated by the higher TAM sensitivity of *Sdhb* ^*fl/fl*^ animals in our colony. It is also possible that the *Sox10* driver is not ideal for targeting susceptible precursor cells without damage to other tissues expressing this pervasive neural crest marker.

We also considered the possibility that the TAM dosage used here resulted only in conversion of cells from *Sdhc* ^*fl/fl*^ to a heterozygous *Sdhc* ^*−/fl*^ condition, not anticipated to initiate tumors. We consider it extremely unlikely that only *Sdhc* ^*−/fl*^ cells were generated in the adrenal medulla because the tested TAM dose was adequate to induce gait defects and fur pigmentation defects in TAM‐treated *Sdhc* ^*fl/fl*^ animals. Neither of these phenotypes has been observed for *Sdhc* heterozygous mice studied in our laboratory. This observation demonstrates that the utilized TAM dose was sufficient to rearrange both floxed *Sdhc* alleles in at least some treated cells in multiple tissues. PPGL tumorigenesis should require only generation of isolated SDHC‐null cells acting as “seeds” within the adrenal medulla. Finally, we continue to consider the possibility that current mouse strain backgrounds contribute unknown tumor suppressor genes that block SDHx‐loss murine PPGL tumorigenesis.

Rather than PPGL tumorigenesis, approximately half of our conditional SDHC‐knockout animals display mild but intriguing developmental defects consistent with metabolic dysfunction of certain neural crest‐derived cells. Future detailed studies will be needed to establish the detailed etiology of these pathologies. Though detailed pathology is unavailable, our observation of an induced hind limb gait abnormality is suggestive of a neural tube closure defect, though overt spina bifida is not observed. The presence of patches of white fur in our conditional *Sdhc*‐loss mice suggests that a oxidative phosphorylation defect in Sox10^+^ neural crest‐derived melanocytes causes clones of nonpigmented cells.

## AUTHOR CONTRIBUTIONS

EPL and FAK performed experiments and analyzed data; NMG, PO‐S, JL‐B, and BW analyzed pathology; IA and LJM designed experiments; EPL and LJM wrote the manuscript.

## CONFLICT OF INTEREST STATEMENT

The authors report no conflicts of interest.

## Supporting information


Figures S1–S3

Video S1

Video S2

Video S3

## Data Availability

The data that support the findings of this study are available in Sections 2 and 3, and/or Supporting Information of this article. Other data are available on request from the corresponding author.
