# Supplementary material for: Mouse developmental defects, but not paraganglioma tumorigenesis, upon conditional Complex II loss in early Sox10+ cells
Source: FASEB Bioadv. 2024 Jul 24;6(9):327–36. doi: 10.1096/fba.2024-00056 (PMC11467736; doi:10.1096/fba.2024-00056)
Supplement: Supplementary file 1 — Figures S1–S3 [file FBA2-6-327-s003.pdf]

Supplemental material for:

Mouse developmental defects, but not paraganglioma tumorigenesis, upon conditional Complex II loss in early Sox10^{+} cells

Elizabeth P. Lewis[1], Fatimah Al Khazal[1], Brandon Wilbanks[1], Naomi M. Gades[2], Patricia Ortega-Sáenz[3], José López-Barneo[3], Igor Adameyko[4], and L. James Maher, III[1]

[1]Department of Biochemistry and Molecular Biology, Mayo Clinic College of Medicine and Science
200 First St. SW, Rochester, MN 55905, USA
[2]Department of Comparative Medicine, Mayo Clinic, 13400 E Shea Blvd, Scottsdale, AZ 85259
[3]Instituto de Biomedicina de Sevilla (IBiS), Hospital Universitario Virgen del Rocio/CSIC/Universidad de Sevilla, Sevilla, Spain.
[4]Department of Physiology and Pharmacology, Karolinska Institutet, Solnavägen 9, Biomedicum, 17165 Solna, Sweden

Correspondence should be addressed to:
Jim Maher
Department of Biochemistry and Molecular Biology
Mayo Clinic College of Medicine and Science
200 First St. SW, Rochester, MN 55905, USA
Phone: 507-284-9041
e-mail: maher@mayo.edu

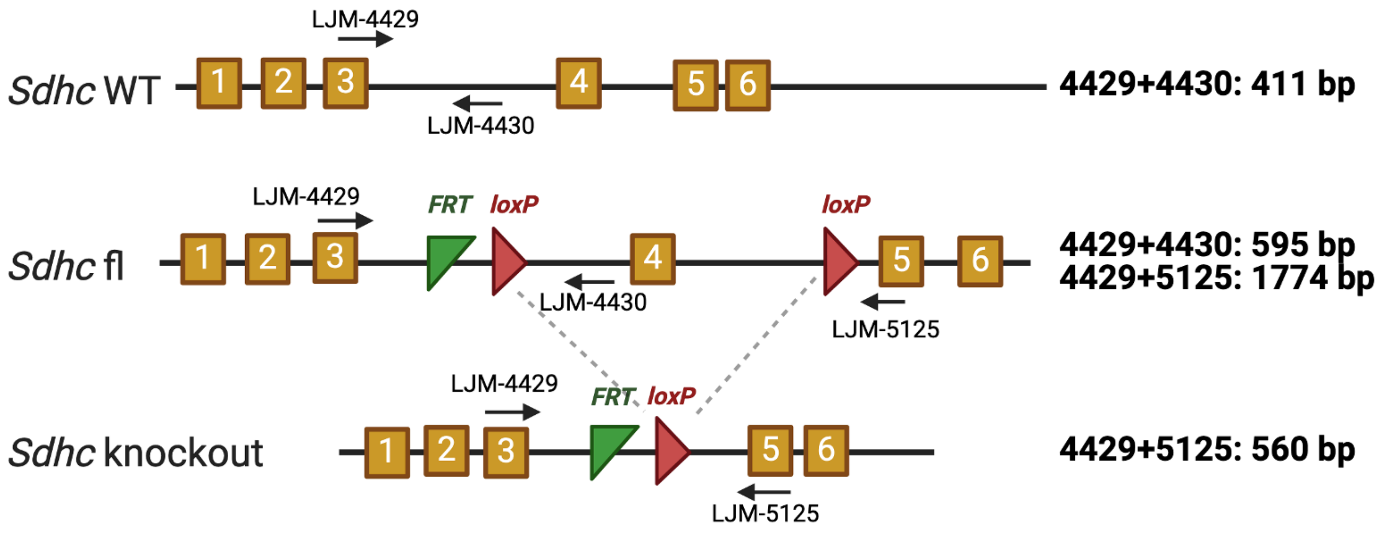

Supplemental figures

Fig. S1. *Sdhc* genotyping strategy. PCR primers are indicated with the prefix "LJM". Diagnostic PCR product lengths are indicated at right. Primer sequences are provided in Methods.

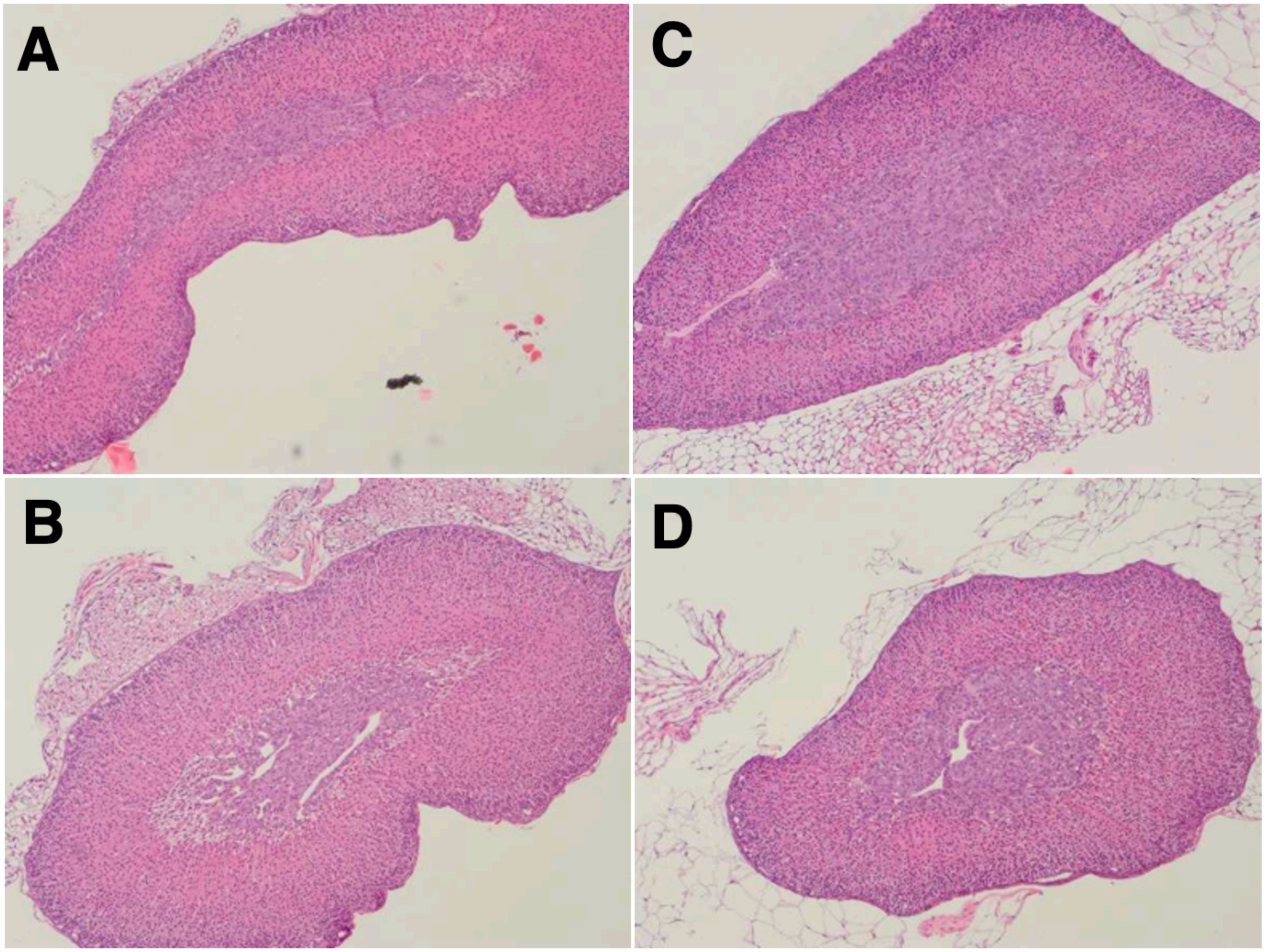

Fig. S2. Examples of adrenal medullae showing normal morphology. A, B: Adrenal medullae from TAM-treated *Sox10::iCreER*^{T2} *Sdhc* $^{fl/fl}$ conditional knockout mice that showed coat pigmentation and gait defects. C, D: Adrenal medullae from TAM-treated *Sdhc* $^{fl/fl}$ mice lacking iCre. All images are tissue sections stained with hematoxylin and eosin and shown at 40× magnification.

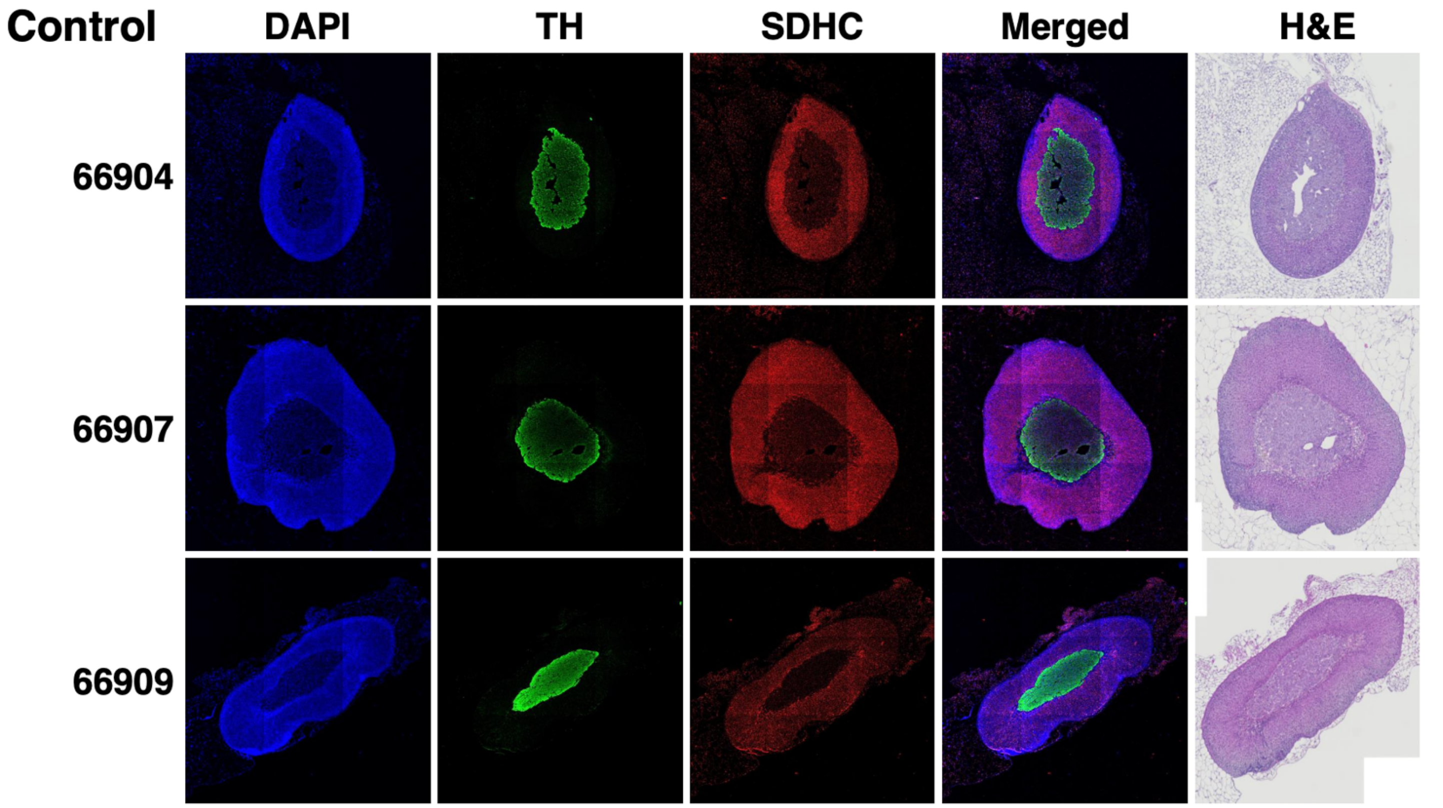
Control
DAPI
TH
SDHC
Merged
H&E
66904
66907
66909

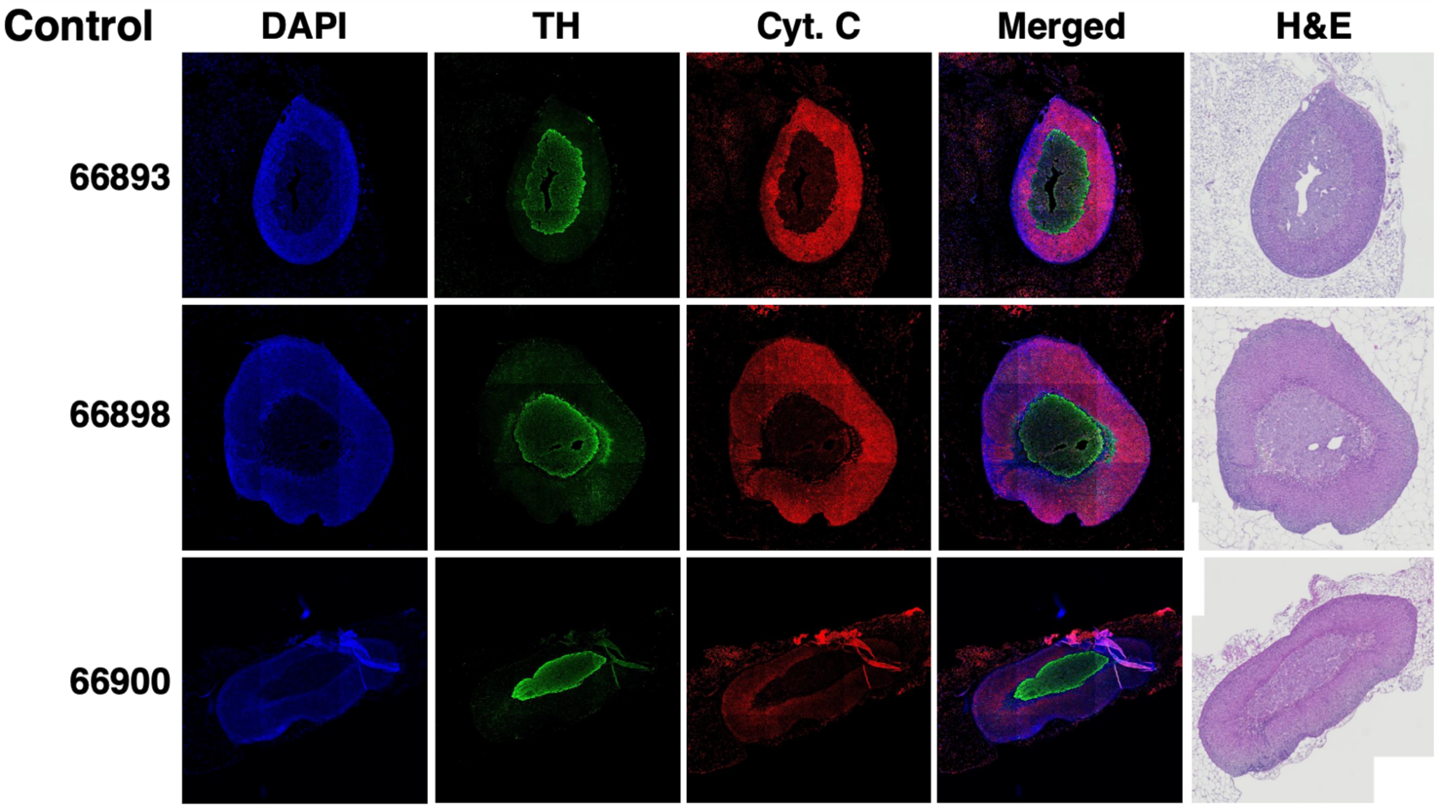
Control
DAPI
TH
Cyt. C
Merged
H&E
66893
66898
66900

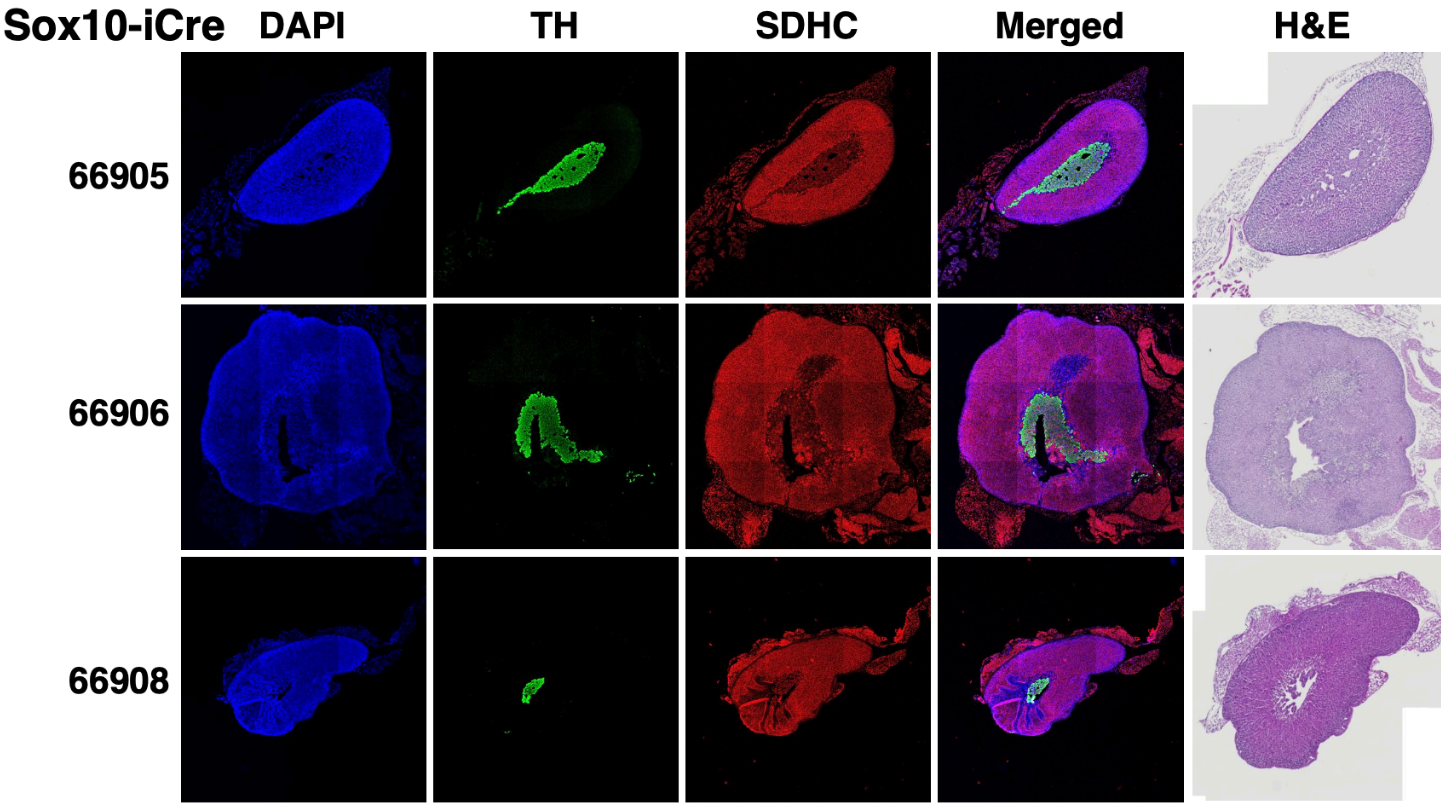
Sox10-iCre
DAPI
TH
SDHC
Merged
H&E
66905
66906
66908

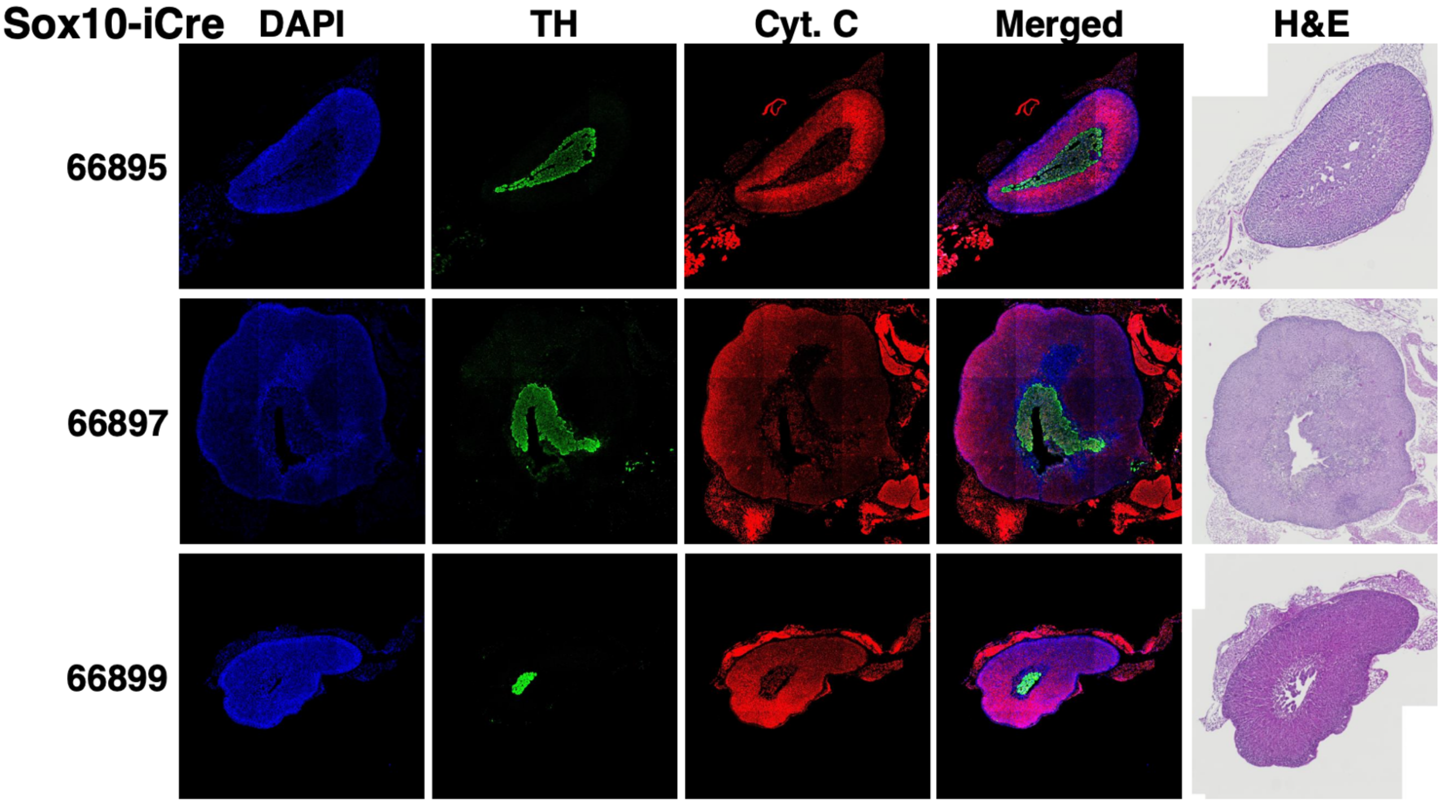
Sox10-iCre
DAPI
TH
Cyt. C
Merged
H&E
66895
66897
66899

Fig. S3. Adrenal glands from three representative TAM-treated *Sox10::iCreER*^{T2} *Sdhc* $^{fl/fl}$ conditional knockout mice that showed developmental phenotypes, and three representative SOX-10-iCre-negative cases without phenotypes. Specimens were sectioned and stained with hematoxylin and eosin and the serial sections stained with anti-tyrosine hydroxylase and anti-cytochrome C or anti-tyrosine hydroxylase and anti-SDHC. Enhanced anti-cytochrome C staining was considered as a surrogate for SDHC loss. All images are tissue sections stained with hematoxylin and eosin and shown at 40× magnification. Indices indicate specimen serial numbers.

Supplemental videos

Supplemental video S1 (video_1.mp4: 32 seconds). Two *Sdhc*$^{fl/fl}$ *Sox10-iCre* animals (note white coat markings and hind limb gait pathology) and one unaffected *Sdhc*$^{fl/fl}$ (no *iCre*) littermate from a mother injected with TAM at E11.5.

Supplemental video S2 (video_2.mp4: 44 seconds). Same cohort as in video 1.

Supplemental video S3 (video_3.mp4: 50 seconds). An individual *Sdhc*$^{fl/fl}$ *Sox10-iCre* animal from a mother injected with TAM at E11.5. Note subtle hind limb gait pathology.
